# Effect of sequence padding on the performance of deep learning models in archaeal protein functional prediction

**DOI:** 10.1038/s41598-020-71450-8

**Published:** 2020-09-03

**Authors:** Angela Lopez-del Rio, Maria Martin, Alexandre Perera-Lluna, Rabie Saidi

**Affiliations:** 1 B2SLab, Department d’Enginyeria de Sistemes, Automàtica i Informàtica Industrial, Universitat Politècnica de Catalunya, 08028 Barcelona, Spain; 2 Department of Biomedical Engineering, Institut de Recerca Pediàtrica Hospital Sant Joan de Dèu, 08950 Esplugues de Llobregat, Spain; 3 European Molecular Biology Laboratory, European Bioinformatics Institute (EMBL-EBI), Hinxton, CB10 1SD UK

**Keywords:** Data processing, Machine learning, Protein analysis, Protein function predictions, Sequence annotation, Computational biology and bioinformatics

## Abstract

The use of raw amino acid sequences as input for deep learning models for protein functional prediction has gained popularity in recent years. This scheme obliges to manage proteins with different lengths, while deep learning models require same-shape input. To accomplish this, zeros are usually added to each sequence up to a established common length in a process called zero-padding. However, the effect of different padding strategies on model performance and data structure is yet unknown. We propose and implement four novel types of padding the amino acid sequences. Then, we analysed the impact of different ways of padding the amino acid sequences in a hierarchical Enzyme Commission number prediction problem. Results show that padding has an effect on model performance even when there are convolutional layers implied. Contrastingly to most of deep learning works which focus mainly on architectures, this study highlights the relevance of the deemed-of-low-importance process of padding and raises awareness of the need to refine it for better performance. The code of this analysis is publicly available at https://github.com/b2slab/padding_benchmark.

## Introduction

Since the breakthrough of deep learning (DL)^1^, deep neural networks are being successfully applied in computational biology^2,3^. This is due to their capacity for automatically extracting meaningful features from raw data^4^. Specifically, DL is useful in the context of biological sequences, such as proteins or RNA, because it can learn directly from the sequence and hence, capture nonlinear dependencies and interaction effects. Some examples of applications of DL on biological sequences include prediction of specifities of DNA and RNA binding proteins^5^, DNA function quantification^6^, de novo peptide design^7^, detection of conserved DNA fragments^8^, prediction of protein associated GO terms^9^ or quantification of the impact of genetic variation on gene regulatory mechanisms^3^. The specific DL architectures able to leverage the inner structure of sequential biological data are Convolutional Neural Networks (CNN) and Recurrent Neural Networks (RNN). CNNs entail translational invariance^10^ and can be used to find relevant patterns with biological meaning^5,8,11,12^. For their part, bidirectional RNNs (and the derived Long Short-Term Memory and Gated Recurrent Units) are appropiate for modelling biological sequences since they are suited for data with a sequential but non-causal structure, variable length, and long-range dependencies^13–16^. Both architectures are usually combined, as in DEEPre^17^, where a CNN-RNN model performs a hierarchical classification of enzymes.

Proteins are long linear sequences constituted by amino acid residues attached covalently. These amino acid residues are represented by letters that cannot be directly processed by the mathematical operations used by DL models. Choosing how to digitally encode amino acids is a crucial step in this context, since it can affect to the overall performance of the models^18^. A comprehensive review and assessment on different amino acid encoding methods^19^ shows that position specific scoring matrix (PSSM), an evolution-based position dependent methodology, achieves the best performance on protein secondary structure prediction and protein fold recognition tasks. However, this type of encoding is very consuming computationally^20^ and its applicability is limited to proteins with known homologous sequences^19^, which could highly decrease the generalisation capabilities of the predictor for non evolutionary related proteins. Traditionally, proteins have also been encoded into feature vectors^21,22^. These encoding features are generally aggregative and not bijective, such as signatures, physicochemical properties or amino acid composition. From aggregative features, the original sequence cannot be recovered, resulting in a loss of protein information.

**Figure 1:**
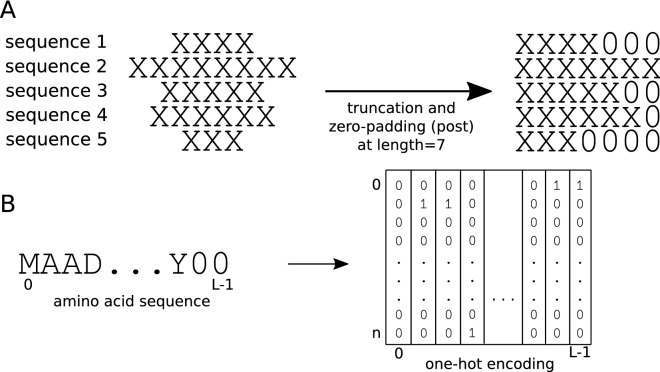
Schematic explanation of one-hot encoding, zero-padding and truncation of amino acid sequences (**A**) Amino acid sequences of different lengths are shaped to the common dimension of 7 by truncating or padding zeros at the end. (**B**) Amino acid sequence at common length *L* is transformed to a binary matrix $(n+1) \times L$, being *n* the number of different amino acids and placeholders. Each column of this matrix is full of zeros, being one only in the position of the corresponding amino acid.

The analogy between text and proteins, understood as sequences of characters with a meaning, has motivated the application of Natural Language Processing (NLP) techniques to amino acid sequences. Along these lines, machine-learning derived embeddings^23–26^ and one-hot encoding^7,9,12,14,17,27^ have become very popular. Specifically, the latter method has been widely used in protein-based DL models since neural networks are able to extract features from raw data. A schematic explanation of one-hot encoding is shown in Fig. 1B. Every amino acid of a protein sequence is represented by a binary vector of length $n+1$, *n* being the number of different amino acids and placeholders. In this vector, all but the corresponding entry for that amino acid is set to zero. As a result, a protein of length *L* is represented by a $(n+1) \times L$ binary matrix.

The main problem of one-hot encoding is that each protein has a different length, while all input vectors should be of the same size to be fed into the model. To overcome this issue, sequence padding and truncation are usually applied^7,9,12,14^. This means establishing a common length for all proteins and then, truncating longer proteins to that length or filling shorter proteins with an “artificial” character up until that length (see Fig. 1A). This process of completing a sequence is called padding and the character used for filling could be any that is not used in the sequences themselves. To this matter, zero character (“0”) is the most commonly used. Padding zeros can be added at any position of the sequence, for example at the N- and C- terminals of the sequences^28^. In practice, they are usually added at the end^7,14^. However, details on the concrete steps of sequences padding are often omitted as they are deemed of low importance for the results of the study^9,12,17,27^. Even when this information is given, there is no proper justification on the padding choice^7,14,28^. This is partly due to the lack of exhaustive studies on the effect of padding the sequences. Up to our knowledge, the work of Reddy et al.^29^ is the only study on the effect of sequence padding on deep learning models. It was applied on a NLP sentiment analysis task and only pre- and post- padding types were tested. Since it is a different application domain and the options they test are limited, a more comprehensive study for the case of biological sequences is needed. Likewise, alternative types of padding to those usually implemented (zeros at the end of the sequence, at the beginning or both) have not been yet explored.

Domains of application involving recurrent neural networks also make use of mask layers, in order to inform the model to skip the padding positions in the objective function and gradients^30^. However, masking lacks general support for convolutional, feed-forward, flatten or pooling layers. Since many of the amino acid sequence models in the literature contain some of these layers^8,9,12,14,27^, and considering that recurrent layers have been proven not to always be the best choice in sequence-based models^31^, it is still important to evaluate the potential effects of padding.

In this paper, we report a systematic analysis on how different types of padding affect to protein-based DL models performance. We evaluate this effect on three different DL architectures: only feed-forward neural networks (only_denses), feed-forward neural networks coupled with a convolutional layer (1_conv) and feed-forward neural networks coupled with a stack of convolutional layers (stack_conv). We also introduce four novel padding types (mid-, strf-, rnd-, and zoom-) and we classify them along with the known types (pre-, post- and ext-) into dense and sparse paddings. Dense paddings are those keeping zeros together in a block (pre- at the beginning, post- at the end, mid- in the middle and ext- at both ends), while in sparse paddings, zeros are interspersed on the sequence (randomly in the case of rnd- and uniformly for strf-) or amino acids are duplicated (zoom-). Finally, we quantify the effect for each type of padding. The chosen task for this study is a hierarchical classification of enzymes with two levels: the first is a binary classification of proteins into enzymes/non-enzymes (task 1), and the second is a multi-label prediction of the enzyme type (task 2).

## Results

### Performance metrics

**Figure 2:**
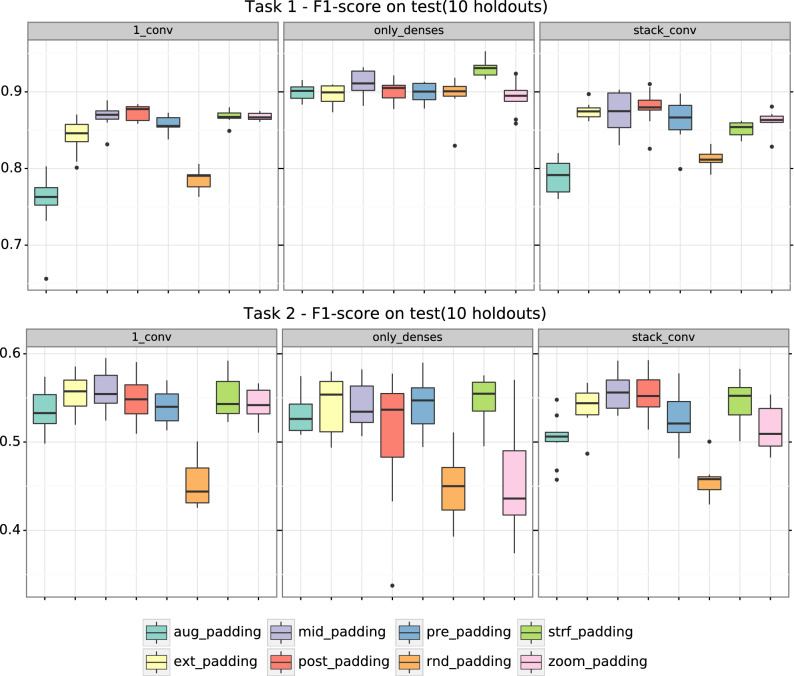
Macro F1-score on test for each type of padding in each tested architecture. Each boxplot comprises 10 data points (which is the number of folds).

A summary of the F1-score (macro average), accuracy and Area under the receiver operating characteristic curve (AUC from now) on test for each architecture, each type of padding and each task (only task 1 in the case of AUC) is shown in Table 1. Since the trends observed for these metrics analogous, we will focus on F1-score. Fig. 2 shows the macro F1-score on test for each type of padding in each of the tested architectures, both for task 1 and task 2. The same figure but for accuracy can be found in Fig. S1 of the Supporting Information. Figures S2–S7 show F1-score results per label (non-enzyme/enzyme in the case of task 1 and 1–7 enzyme types in the case of task 2) for each task and each of the architectures.

**Table 1:** Summary of F1-score (macro average), accuracy and AUC on the test set. Results are reported for all the different types of padding for both task 1 and task 2 in each one of the tested architectures (except AUC, which is only available for Task 1). Mean ± standard deviation of the 10 folds.

	Padding type	1_conv	only_denses	stack_conv
Task 1 F1-score	aug	$0.756 \pm 0.041$	$0.900 \pm 0.011$	$0.790 \pm 0.022$
	ext	$0.842 \pm 0.022$	$0.896 \pm 0.013$	$0.875 \pm 0.010$
	mid	$0.868 \pm 0.016$	$0.911 \pm 0.018$	$0.874 \pm 0.026$
	post	$0.873 \pm 0.010$	$0.900 \pm 0.014$	$0.879 \pm 0.024$
	pre	$0.858 \pm 0.011$	$0.899 \pm 0.013$	$0.863 \pm 0.028$
	rnd	$0.786 \pm 0.014$	$0.896 \pm 0.025$	$0.812 \pm 0.011$
	strf	$0.867 \pm 0.008$	$0.930 \pm 0.011$	$0.851 \pm 0.010$
	zoom	$0.868 \pm 0.005$	$0.893 \pm 0.021$	$0.862 \pm 0.014$
Task 2 F1-score	aug	$0.536 \pm 0.025$	$0.531 \pm 0.022$	$0.504 \pm 0.026$
	ext	$0.554 \pm 0.022$	$0.543 \pm 0.034$	$0.540 \pm 0.023$
	mid	$0.558 \pm 0.021$	$0.542 \pm 0.027$	$0.557 \pm 0.024$
	post	$0.550 \pm 0.025$	$0.509 \pm 0.075$	$0.554 \pm 0.024$
	pre	$0.541 \pm 0.020$	$0.541 \pm 0.030$	$0.527 \pm 0.028$
	rnd	$0.452 \pm 0.026$	$0.448 \pm 0.034$	$0.455 \pm 0.020$
	strf	$0.550 \pm 0.024$	$0.548 \pm 0.026$	$0.547 \pm 0.024$
	zoom	$0.543 \pm 0.019$	$0.456 \pm 0.063$	$0.515 \pm 0.026$
Task 1 Accuracy	aug	$0.758 \pm 0.037$	$0.901 \pm 0.011$	$0.790 \pm 0.023$
	ext	$0.843 \pm 0.023$	0.$896 \pm 0.014$	$0.875 \pm 0.010$
	mid	$0.868 \pm 0.016$	0.$911 \pm 0.018$	$0.874 \pm 0.027$
	post	$0.873 \pm 0.010$	0.$900 \pm 0.014$	$0.879 \pm 0.024$
	pre	$0.858 \pm 0.011$	0.$899 \pm 0.013$	$0.863 \pm 0.028$
	rnd	$0.790 \pm 0.013$	0.$897 \pm 0.025$	$0.815 \pm 0.011$
	strf	$0.868 \pm 0.008$	0.$930 \pm 0.011$	$0.852 \pm 0.010$
	zoom	$0.869 \pm 0.005$	0.$893 \pm 0.021$	$0.863 \pm 0.014$
Task 2 Accuracy	aug	$0.548 \pm 0.009$	$0.536 \pm 0.012$	$0.527 \pm 0.015$
	ext	$0.539 \pm 0.016$	$0.544 \pm 0.025$	$0.549 \pm 0.017$
	mid	$0.539 \pm 0.017$	$0.545 \pm 0.018$	$0.569 \pm 0.011$
	post	$0.530 \pm 0.011$	$0.532 \pm 0.024$	$0.560 \pm 0.016$
	pre	$0.532 \pm 0.009$	$0.542 \pm 0.016$	$0.545 \pm 0.018$
	rnd	$0.455 \pm 0.011$	$0.473 \pm 0.020$	$0.509 \pm 0.017$
	strf	$0.556 \pm 0.021$	$0.556 \pm 0.012$	$0.565 \pm 0.015$
	zoom	$0.550 \pm 0.014$	$0.528 \pm 0.021$	$0.531 \pm 0.010$
Task 1 AUC	aug	$0.859 \pm 0.021$	$0.951 \pm 0.010$	$0.891 \pm 0.013$
	ext	$0.927 \pm 0.011$	$0.966 \pm 0.003$	$0.949 \pm 0.005$
	mid	$0.945 \pm 0.009$	$0.972 \pm 0.006$	$0.952 \pm 0.010$
	post	$0.945 \pm 0.007$	$0.969 \pm 0.003$	$0.956 \pm 0.007$
	pre	$0.935 \pm 0.006$	$0.967 \pm 0.004$	$0.949 \pm 0.008$
	rnd	$0.871 \pm 0.011$	$0.946 \pm 0.014$	$0.891 \pm 0.009$
	strf	$0.939 \pm 0.006$	$0.978 \pm 0.003$	$0.927 \pm 0.006$
	zoom	$0.937 \pm 0.004$	$0.978 \pm 0.005$	$0.944 \pm 0.005$

According to Table 1 and Fig. 2, although the different architectures seem to have similar F1-score values, only_denses architecture is the one that achieves the best performance for task 1 (paired Wilcoxon test, two-sided, $\hbox {p}=9\mathrm {e}{-15}$ vs 1_conv and $\hbox {p}=4\mathrm {e}{-13}$ vs stack_conv). Regarding task 2, we can see at Fig. 2 that the trend is not as clear as in task 1. 1_conv has the best performance (paired Wilcoxon test, two-sided, $\hbox {p}=9\mathrm {e}{-4}$ vs only_denses, $\hbox {p}=2\mathrm {e}{-5}$ vs stack_conv), while there are no statistical differences between stack_conv and only_denses.

Regarding metrics per label, in task 1 (Figs. S2, S4 and S6) best recall results for non-enzymes were achieved in convolutional architectures, but the opposite trend is shown for the baseline architecture (only_denses). For task 2, classification of enzyme types 1, 4 and 6 achieved lower performance than 2 and 5. This applies to the three architectures (Figs. S3, S5 and S7). As for enzyme type 7, results show high variability in comparison with the other types due to the limited number of samples of this class.

### Effect on input space

**Figure 3:**
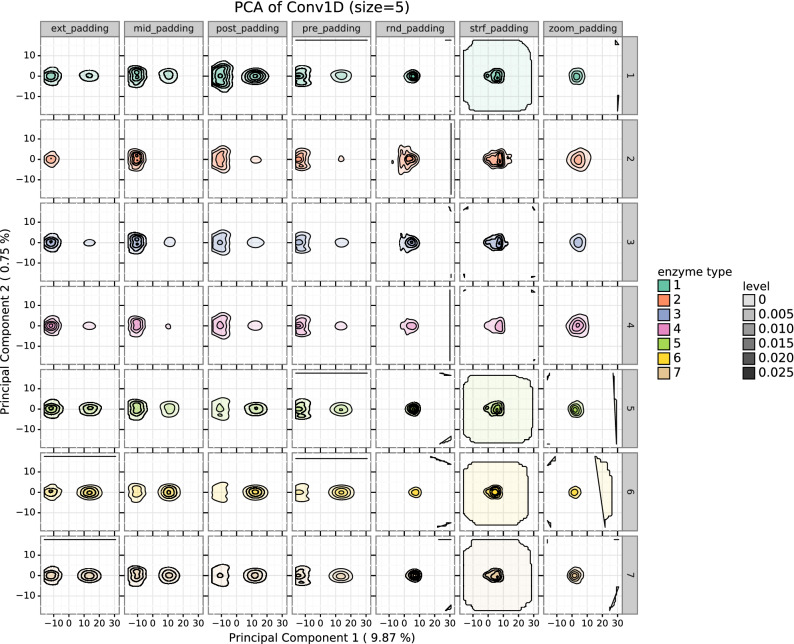
Density representation of the PCA of the activations of the convolutional layer. Figure shows PC1 vs PC2 of the activations of the 1D Convolutional layer for the 1_conv model in task 2, for the 10 folds. This representation comes from applying a PCA to the convolutional filter activations after the prediction of 14 enzymatic sequences of different EC number using each padding type. Then, the graphical representation was stratified by padding and enzyme type. We can see that according to the structure of the distributions, there seems to be two different groups of enzymes: 2, 3, 4 and 1, 5, 6, 7. Regarding types of padding, the activations for dense paddings are similar between them (two clusters separated along PC1) and different from sparse paddings.

We studied the distribution of the activations of the 1D Convolutional layer for the 1_conv model to analyse the effect of the padding type in the input space by means of a Principal Components Analysis (PCA). Figure 3 displays the density plot showing the principal components (PC) 1 and 2 of the activations from the 1D convolutional layer of the 1_conv architecture for each type of padding on each fold in task 2. In Fig. S8 of the Supporting Information, the same representation for task 1 is shown. Focusing on Fig. 3 for task 2, the distribution of the activations is very similar for dense types of padding (ext-, mid-, post- and pre-). These dense activations are grouped in two clusters separated along the PC1. Sparse paddings (rnd-, strf-, zoom-) activations have a distribution very different to that from dense paddings. In this case, activation points are condensed in one area, although each one of these types of padding has its own structure. Regarding enzyme types, according to the structure of the distributions, there seems to be two different groups: enzyme types 2, 3 and 4 are very similar between them and in turn, different to types 1, 5, 6 and 7. Table S7 of the Supporting Information quantifies the effect of the enzyme and the padding types on the PC1 of the activations using a linear model. All the terms of the model are significant.

### Explanatory models

We used linear models to further explain the performance metrics and the effect of different variables (padding type, enzyme type, architecture) to the DL models behaviour. These explanatory models were also used to address specific questions regarding the effect of padding.

The full additive linear model in Eq. (1) describes the F1-score values on test and it is shown on the Table S1 of the Supporting Information. It shows that some types of padding have indeed an effect on models performance, both for task 1 and 2. For example, for all the architectures (since it is an additive model) in task 1, aug-, pre-, ext- and rnd- have worse performance when compared to the reference padding type (post-) (*p* value $< 0.05$). In the same setting but for task 2, rnd- and zoom have significantly worse performance than post- (*p* value $<0.01$).

**Figure 4:**
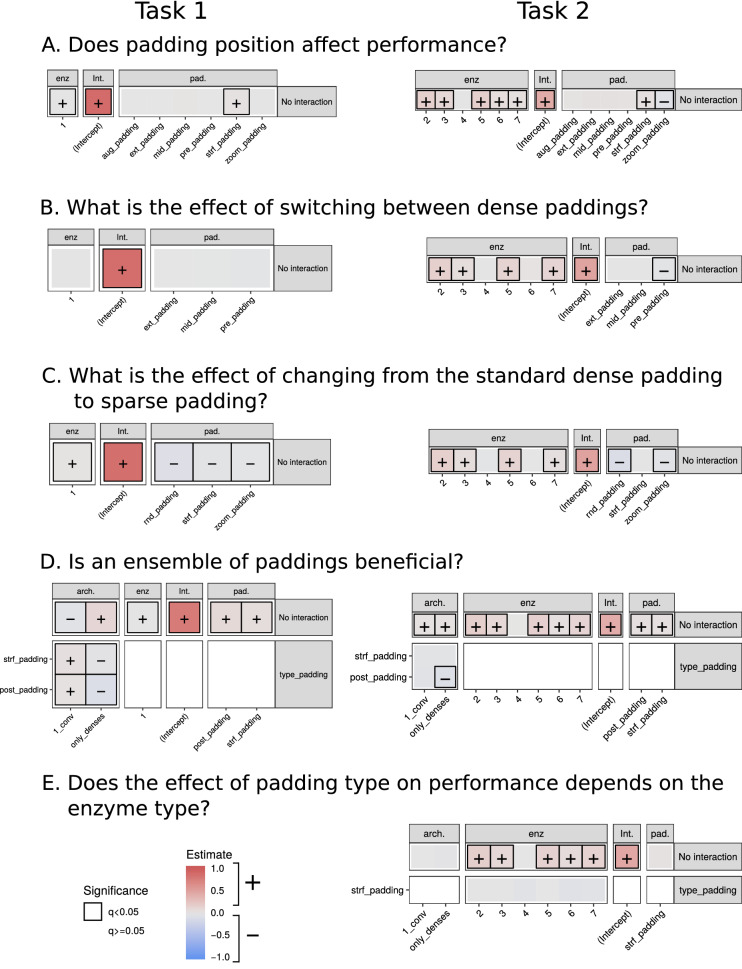
Linear models on F1-score for both tasks 1 and 2 focusing on five specific questions. The “No interaction” facet represent estimates of additive terms of the model, while the other facet represent the interaction between two factors. Models regarding questions A, B and C have no interaction terms and thus, they only have the “No interaction” facet. Only coloured tiles correspond to model coefficients; the white ones are outside the model specification. The colour of each category of a term represents the value of the estimate: red tiles correspond to positive estimates, specified with a “+”, blue tiles correspond to negative estimates, specified by “−” and grey tiles are close to zero. Framed categories are those that have a significant effect on each question (adjusted *p* value $< 0.05$). An example on how to interpret this figure: in Model B task 2, switching from post_padding (reference) to pre_padding in stack_conv (reference) implies a decrease in performance.

Figure 4 and Tables S2–S6 gather the answers to our specific questions on the effect of padding on the different architectures and enzyme types. The colour represents in each case the difference between each category and the reference category of that factor. The sign of the corresponding estimate is represented if that difference is statistically significant. The constant term of a model (Intercept) shows the prediction when all the categorical variables have their reference values.

### A. Does padding position affect performance?

Figure 4A and Table S2 show that the classification performance of the baseline model (only_denses) for task 1 is the same for all the types of dense padding, except for strf-padding, which is better than post- (predicted F1-score of 0.926 vs 0.896). In the same way for task 2, only strf- significantly outperforms post-padding (0.472 vs 0.432), while zoom- has a worse performance (0.379).

### B. What is the effect of switching between dense paddings?

Figure 4B and Table S5 for task 1 show that for stack_conv, there are no differences in performance between dense paddings. Regarding task 2, only pre-padding is significantly worse than post-padding (0.457 vs 0.484) for stack_conv.

### C. What is the effect of changing from the standard dense padding to sparse padding?

Figure 4C and Table S4 show that for stack_conv in both tasks, sparse paddings have significantly worse performances than dense paddings. For task 1, post- significantly outperforms rnd-, strf- and zoom-padding (predicted F1-scores of 0.868 vs 0.801, 0.840 and 0.851, respectively). For task 2, post- also outperforms rnd- and zoom-padding (0.498 vs 0.400 and 0.460, respectively).

### D. Is an ensemble of paddings beneficial?

We tested for the three architectures if data augmentation regarding padding (aug-padding) improved the performance when compared to post-padding (representing the dense paddings) and strf-padding (representing the sparse paddings). To quantify the changes, we used aug-padding as reference level in padding type. Figure 4D and Table S5 show that both post- and strf- significantly outperform aug-padding both for task 1 and task 2.

For task 1, with the baseline architecture (stack_conv) aug-padding gets the worst predicted F1-score (0.786), while it is 0.875 and 0.847 for post- and strf- respectively. For the reference padding type (aug-), the stack_conv architecture performs worse than only_denses (0.785 vs 0.895) but better than 1_conv (0.785 vs 0.752). Interactions show that both strf- and post- have a more positive effect on performance with respect to the baseline (aug-) for 1_conv (0.863 and 0.868 vs 0.752 from aug-) than for stack_conv (0.847 and 0.875 vs 0.786). On the contrary, changing from aug- to strf- and post- have less performance improvement for only_denses (0.895 from aug- vs 0.926 and 0.896) than for stack_conv (0.786 vs 0.847 and 0.875), although even so only_denses still outperforms stack_conv.

Regarding task 2, with the baseline architecture aug-padding also gets the worst performance when compared to post- and strf-padding (0.421 vs 0.471 and 0.464). For baseline padding type, both 1_conv and only_denses significantly outperform stack_conv (0.453 and 0.449 vs 0.421). Interactions show that 1_conv reacts the same way to changes of padding type than stack_conv. But in the case of only_denses, changing from aug- to post-padding (0.449 vs 0.426) has less performance improvement than for stack_conv (0.421 vs 0.471), causing aug- to outperform post-.

### E. Does the effect of padding type on performance depends on the enzyme type?

We checked for all the types of architectures and for dense and sparse paddings (represented by post- and strf-, respectively) the effect of enzyme type on model performance (only for task 2). Results (Fig. 4E and Table S6) show that for both padding types, the performance for enzymes with the first EC number digit 2 (0.619), 3 (0.555), 5 (0.598), 6 (0.532) and 7 (0.597) is better than for digits 1 (0.457) and 4 (0.454). Interactions are not significant, meaning this trend applies to all the architectures. This is consistent with the results of the previous questions, where enzyme types 4, 1 and sometimes 6 are shown to decrease performance.

## Discussion

It is not the aim of this paper to study differences on performance between architectures. However, in general terms only_denses has shown to achieve the best performance for task 1 while both convolutional architectures work better for task 2 (see Tables 1, S1–S4 and S6, and Fig. 2-4). Quantitatively, for the full additive model (eq. 1) in task 1, only_denses get a predicted F1-score of 0.916 vs 0.864 of stack_conv and 0.853 of 1_conv for the baseline post-padding type. Using the same model for task 2, predicted F1-score of only_denses is 0.444 versus 0.454 of stack_conv and 0.464 of 1_conv. The reason why only_denses is the best architecture for task 1 could be that the task of classifying amino acid sequences between enzymes and non-enzymes is more related to the presence/absence or count of certain amino acids than to their position within the sequence. In other words, if we could consider them to be amino acid sets instead of sequences as it happens in other fields^32^. On the contrary, classifying enzymes into their types is a more complex task that might imply amino acid patterns and position information, thus a convolutional architecture is a better choice.

Along the same lines, we have seen that task 1 has a better performance than task 2 for all the architectures (Table 1 and Fig. 2). F1-score ranges from $0.756 \pm 0.041$ to $0.930 \pm 0.011$ for task 1, while for task 2 it is comprised between $0.448 \pm 0.034$ and $0.558 \pm 0.021$. Task 1 results are similar to those obtained by DEEPre^17^ for their equivalent Level 0 prediction, but results obtained for task 2 are worse than their report for Level 1. This was expected, since we use the same architecture for a simple binary classification and for a multi-class classification problem. A more complex, optimized model may improve the performance for the first digit prediction problem, but this was out of the scope of this study. We chose the architectures of both tasks to be as simple, comparable and interpretable as possible.

We have confirmed that padding type has an effect on model performance (see Tables S1–S6 and 1, Fig. 4). In Fig. 3 and S8 we could see that indeed, models reflect differences for each type of padding in their input space.

In general, there are no differences between dense paddings (see Fig. 4A,B and Tables S2–S3), neither for convolutional nor for only_denses architectures. This applies for both task 1 and task 2, although for the latter pre-padding underperforms the rest of dense paddings (predicted F1-score 0.457 from pre- vs 0.484 from post-). Therefore, dense paddings are interchangeable for fully feed-forward and dense architectures and we could stick to the default option (post-padding).

There are differences between sparse paddings. For the baseline model (only_denses) in both tasks (Fig. 4A and Table S2), strf_padding has shown to outperform the rest of the paddings: for task 1, strf- has a predicted F1-score of 0.926 vs 0.896 from post-; in task 2, strf- has an estimate of 0.472 while for post- it is 0.432; macro-average for the F1-score on test is 0.930 ± 0.011 for task 1 and 0.548 ± 0.034 for task 2 (Table 1) . This might be because feed-forward neural networks are position-sensitive and moving a block of zeros along the sequence (as in different types of dense padding) can alter the way the networks process them. Strf- does not comprise a block of zeros, but they are spread uniformly along the sequence. This distribution seems to compensate this position sensitivity by aligning certain relative positions of the protein where the model might be detecting abundance changes.

On the contrary, this improvement of performance caused by strf-padding does not apply for stack_conv architecture (Fig. 4C and Table S4). In this case, all sparse paddings perform worse than the baseline post-padding (except for strf- in task 2): for task 1, the predicted F1-score of post-padding is 0.868 vs 0.840, 0.801 and 0.851 from strf-, rnd- and zoom- respectively; for task 2, predicted F1-score is 0.498 for post- vs 0.400 and 0.459 for rnd- and zoom-. Thus, convolutional models works better with dense paddings than with sparse ones.

The differences in activations of the convolutional layers in Fig. 3 further support the classification of paddings into dense and sparse and are in line with the results that we have just reported. The activations for the dense paddings showed to be very similar between them. This is expected due to the translational invariance of the convolutional layers^10^: if zeros are kept together they should be processed in the same way by the convolutional layers, no matter where they are located. In turn, the activations of dense paddings are very different from the sparse ones (Fig. 3). Sparse paddings have also a similar structure, where the activations are condensed in only one centered group.

We have also tested if data augmentation regarding padding (i.e. artificially increasing the size of a dataset by representing one protein by different possibilities of the padded one-hot encoded amino acid sequence) improved model performance as in image deep learning models^33^. Our results (Fig. 4D and Table S5 of the Supporting Information) have shown that aug-padding underperforms dense and sparse paddings both for fully dense and convolutional architectures and for both tasks: for stack_conv task 1 aug- achieves a predicted F1-score of 0.786 vs 0.875 and 0.847 from post- and strf-, respectively; for task 2, 0.421 from aug- vs 0.470 from post- 0.464 and from strf-. In Fig. 2 it also shows to have the worst performance in both tasks for convolutional architectures. Hence, an ensemble of mixed dense and sparse paddings does not improve the performance of the models in this case. Augmented data using only sparse paddings or only dense paddings might work better, because then sequences would be in similar activation spaces.

We observed that models underperformed in enzyme types 1 (oxidoreductases) and 4 (lyases). This was noticeable by displaying the raw metrics (Figs. S3, S5 and S7) and further confirmed through the explanatory models (Fig. 4 and Table S6, the predicted F1-scores for enzymes 2, 3, 5, 6 and 7 are 0.619, 0.555, 0.598, 0.532 and 0.597, respectively, while it is 0.457 and 0.454 for 1 and 4). It does not seem to be related to the number of samples (Fig. S9 of the Supporting Information), to sequence length (see Fig. S10 of the Supporting Information) or to the distribution of the activations (Fig. 3). Therefore, we assume that this is caused because these enzyme types are inherently more difficult to classify, as it happens in^34^. EC number prediction can be challenging in some cases due to divergent evolution (two enzymes with a completely different EC may actually be very similar in sequence)^35^ and parallel evolution of enzyme function (two completely unrelated enzymes catalyse the same reaction and thus, share EC number)^36^.

In Fig. 3 there also seems to be two groups of enzymes according to the distribution of the activations: 1, 5, 6 and 7 vs 2, 3 and 4. This could be partly related to the sequence length: Fig. S10 of the Supporting Information show that enzyme types 2, 3 and 4 are shorter than 1, 5, 6 and 7 (p $=9\mathrm {e}{-54}$ for Mann–Whitney–Wilcoxon test for independent samples, two-sided); moreover, these differences are not so visible for zoom_padding, for which models cannot count zeros. On the other hand, Table S7 of the Supporting Information reports negative coefficients for enzyme types 2, 3 and 4, and positive coefficients for enzymes 5, 6 and 7 (enzyme type 1 is the reference) in the explanatory linear model for PC1, which further supports this grouping.

The results of this study have been obtained for amino acid sequences. It would be needed as a future work to investigate if this effect of padding on model performance can be translated to other biological sequences that are also one-hot encoded and padded, such as RNA^37,38^ and miRNA^39^ or DNA sequences^6^.

## Conclusion

The effect of padding amino acid sequences when they are one-hot encoded had not been comprehensively addressed in the literature yet. The lack of this analysis has caused numerous studies to disregard this step, most of the times taking the “default” option and in some cases, even omitting the details around it. In this paper, we have shown that padding position has an effect on model performance.

We have tested seven types of padding using three different deep learning architectures in a hierarchical enzyme classification problem. It is the first study analysing the relevance of padding one-hot encoded amino acid sequences and its impact on the performance of the studied task.

Our results show that padding the amino acid sequence has an effect on the performance of models. Therefore, more attention should be given to this often omitted step of data pre-processing when building deep learning models for one-hot encoded proteins.

We propose and analyse novel ways of padding proteins when one-hot encoding them for machine learning models (strf-, zoom-, rnd-, mid-). Up until our knowledge, these types have been neither mentioned in the literature nor implemented and made publicly available. We provide the code for their application (https://github.com/b2slab/padding_benchmark), since we have shown that some of them could be more suited for their specific task or architecture.

Our results on EC number classification show that there are no differences between dense paddings. Thus, we can stick to the traditional post-padding, which has proved to outperform the other padding types for convolutional architectures. Regarding sparse paddings, our newly proposed strf-padding has shown to be the best choice for fully feed-forward neural networks, outperforming both dense paddings and the other types of sparse paddings. Lastly, data augmentation regarding the padding (aug-padding) does not improve performance. In contrast, it seems to add noise that causes performance to decrease.

This analysis has been applied to the specific task of EC number prediction. Although we cannot extrapolate these results to other tasks or other deep learning architectures, this is a starting point that highlights the need to avoid neglecting the padding step when one-hot encoding amino acid sequences, since we have shown that it has an effect on model performance.

## Material and methods

### Material

Different types of padding were evaluated on the UniprotKB/Swiss-Prot database^40^ (version 2019_05) protein entries for taxonomy Archaea. For computational reasons we established an upper threshold of 1,000 amino acids for sequence length, leaving 19,385 proteins for training the models (more than 99% of the original entries). For the enzyme classification task performed for the padding analysis, Enzyme Commission number (EC number) annotation was used. EC number is a numeric classification schema for enzymes related to the chemical reactions they catalyze. Each EC number is constituted by 4 numbers separated by dots, being each one a progessively more specific classification. We only used the first digit of the EC number, which refers to the class of enzyme (1:oxidoreductases, 2: transferases, 3: hydrolases, 4: lyases, 5:isomerases, 6: ligases and 7: translocases) and considered the entries without EC number annotation as non-enzymes. 214 entries with more than one EC number were expanded as additional samples, having a total of 19,599 samples. Table 2 shows the enzyme type distribution of the dataset and Fig. S9 of Supporting Information represents this distribution. Data was divided 70/15/15% in training, validation and test sets. The training set was used to fit the model, the validation set was used to evaluate the model fit in each epoch and tune hyperparameters accordingly, and the test set was used to externally evaluate the final model fit. To check the consistency of the results, this splitting was randomly performed 10 times, so each model was trained and tested in each one of these data partitions.

**Table 2:** Distribution of UniprotKB/Swiss-Prot database proteins for taxonomy Archaea. Distribution is shown according to the enzyme type, which is determined by the first digit of the EC number. Entries without EC number are considered as non-enzymes.

	**Non-enzymes**	8,727
	**Total enzymes**	10,872
Enzyme type	1	1,187
	2	3,843
	3	2,123
	4	1,281
	5	603
	6	1,715
	7	120

### Amino acids encoding and protein padding

**Figure 5:**
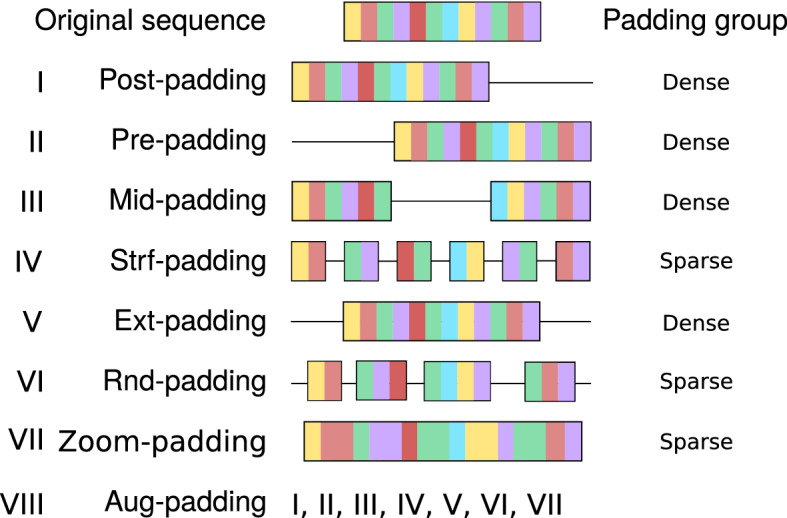
Different types of padding analysed in this study. Each color bin represents an amino acid. Black lines represents zero padding.

Amino acids were represented by one-hot encoding. Seven different padding types were applied to those sequences shorter than 1000 amino acids (see Fig. 5): (I) **post-padding**, adding zeros at the end of the sequences; (II) **pre-padding**, adding zeros at the beginning of the sequence; (III) **mid-padding** (middle), adding the zeros in the middle of the sequence; (IV) **strf-padding** (stratified), distributing the zeros uniformly across the sequences; (V) **ext-padding** (extreme), adding zeros at both ends of the sequence in a balanced way (half of the padding pre- and half of the padding post-); (VI) **rnd-padding** (random), adding zeros at random positions of the sequence; (VII) **zoom-padding**, similar to stratified padding but instead of zeros, contiguous amino acids are repeated; this is the only padding type that “modifies” the sequence length. Additionally, (VIII) **aug-padding** (augmented) will assess the use of data augmentation regarding padding: each sequence will be represented by the seven different padding strategies.

We divided the types of padding in two groups: (1) **dense paddings**, those strategies that keep the sequence to a great extent, i.e. post-padding, pre-padding, ext-padding, mid-padding and (2) **sparse paddings**, which comprises those types of padding which repeatedly modify the structure of the sequence by inserting elements in between: strf-padding, rnd-padding and zoom-padding.

### Classification task: hierarchical models

**Figure 6:**
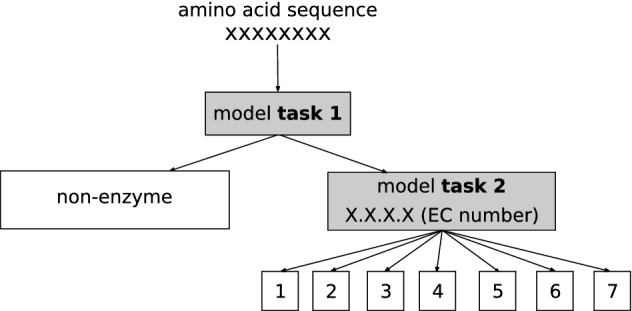
Hierarchical enzyme classification with two levels. Task 1 classifies enzyme/non-enzyme and if enzyme, task 2 classifies by the first digit of the EC number.

We tackled the enzyme classification task as a hierarchical problem with a level-by-level prediction strategy, as in^17^ (see Fig. 6), although we only approached the first two levels of the structure. This decision was taken due to the data imbalance (see Fig. S9 on the Supporting Information and Table 2) between non-enzymes and the less populated enzyme classes (e.g. class 7). We built two prediction models. Firstly, a binary classification model that, given a sequence, predicts if it is an enzyme or not. From now on, it will be referred as *task 1*. Secondly, a multilabel classification model with seven outputs that, given a sequence classified as enzyme by the first model, predicts the class of the enzyme (the first digit of the EC number). This will be referred as *task 2*.

### Models architecture

We analysed the padding effect on three DL architectures: 1. a model with only feed-forward neural networks (it will be referred as *only_denses*), 2. a model with feed-forward neural networks and one 1D convolutional layer (*1_conv*) and 3. a model with feed-forward neural networks and five 1D convolutional layers stacked in parallel (*stack_conv*). The schematic representation of the four models can be found in Figs. S11 and S12. CNNs from the second and third model are aimed to detect meaningful patterns in the amino acid sequence. In all cases, dropout is used to prevent overfitting^41^. The *only_denses* model was considered as baseline or reference model, to have the simplest reasonable deep learning model to which we could compare against^42^ ; the *1_conv* model was chosen to study the effect of adding a convolution to the model and *stack_conv* was taken to check the effect on a convolutional architecture of relative complexity^14,27^. Further details of the models and the corresponding hyperparameters can be found in the Supporting Information file.

We tried to fit a bi-LSTM model to also test the effect of padding on this architecture. However, this model was too complex to converge within the range of parameters of the other three architectures (number of epochs, optimizer, learning rate). As stated by Li et al.^43^, LSTMs have convergence issues when training long sequences (length $\ge$ 1000). Because of this, we considered that the results of the bi-LSTM were not comparable to those from the other architectures and thus, decided to remove it from the analysis.

### Implementation

Models were trained with an Adam optimizer^44^ for 200 epochs, with a batch size of 54 ($\hbox {learning rate} =1\text {E-}4$, $\beta _1 = 0.9$, $\beta _2 = 0.999$). Models were implemented in Python (Keras^45^ 2.2.4 using as backend TensorFlow^46^ 1.8.0) and run on the GPU NVIDIA TITAN Xp and NVIDIA GeForce GTX 1070.

### Performance metrics

The final model is that of weights corresponding to the epoch with the maximum validation accuracy in each case. Accuracy is the proportion of correct predictions. We tested each selected model on the corresponding test set of that data partition. For evaluating and comparing the performance of the different padding types, accuracy, F1-score for each label and macro F1-score on the test set were used. F1-score is the harmonic mean of precision (proportion of positive class predictions that actually belong to the positive class) and recall (proportion of correct positive class predictions out of all positive examples in the dataset). The macro F1-score is computed by calculating F1-score for each label and getting their unweighted mean, hence being insensitive to class imbalance. AUC was also computed for task 1 since it is a binary classification problem. AUC represents the probability that the classifier will rank a randomly chosen positive sample higher than a randomly chosen negative sample. Further details on the definition of these metrics can be found in the Supporting Information file. AUC was computed from raw predicted probabilities, while F1-score and accuracy were calculated from the binarized predictions at threshold 0.5. To statistically compare these metrics between architectures and types of padding, nonparametric two-sided Wilcoxon tests for paired samples were carried out^47^.

### Effect on input space

To analyse the effect of the padding type in the input space, we studied the distribution of the activations of the 1D Convolutional layer for the 1_conv model. This layer has sixty-four filters of size 5 (see Fig. S5 of the Supporting Information).

We randomly selected seven proteins of each type (for task 1) and two proteins of each type (for task 2) from the test set. For the two tasks separately, for each type of padding (except aug_padding) and each fold, we used the final model to predict on those proteins. We extracted the activations of the 1D convolutional layer for each prediction and separated each one of the sixty-four filters as a different sample. This resulted in a matrix of dimensions $64 \times 1000$ representing the activations for each prediction. Stacking the activation data of all the predictions (10 folds $\times 7$ types of padding $\times 14$ enzymatic sequences $= 980$ activations matrices of size $64 \times 1000$) separatedly for each task, we performed a PCA to study and compare the distribution of these activations.

### Explanatory models

The performance metrics were further described through linear models built upon different variables that could affect to the model behaviour. These explanatory models have already been used for similar purposes^14,48^ and provide a way of statistically quantifying and comparing the relevance of the considered variables on the models performance.

Differences in performance between the different types of padding for both tasks were explained and tested in terms of the following linear model:

1 $$\begin{aligned} \hbox {F}1 \sim \hbox {architecture} + \hbox {enzyme}\_\hbox {type} +\hbox {type}\_\hbox {padding} \end{aligned}$$

This full additive model was used as a snapshot of the general contribution of each factor to the F1-score. Reference category for enzyme type was 0 (non-enzyme) for task 1 and enzyme type 1 for task 2, only_denses for architecture and post_padding for padding type. However, to answer more specific questions about the effect of padding on the different architectures and enzyme types, we built more precise, appropriate models in each case (Eqs. (3), (2), (4)). Some of them included an interaction term (represented by *var*1 : *var*2) to check if the effect of *var*1 on the F1-score depends on the value of *var*2. For example, adding $type\_padding:architecture$ would let us identify those cases where the effect of changing the type of padding is different between architectures. Reference category for enzyme type was still 0 for task 1 and enzyme type 1 for task 2, but for architecture and padding type it varies in each case. Table 3 summarizes the questions addressed through the explanatory models, their equations and the reference levels in each case. We considered only_denses as reference in Question A because it is the baseline model and we aimed to check if in this case, different paddings affect differently to model performance. In questions B-E, stack_conv is chosen as reference architecture since it is the more complex and thus, the closest to the state of the art. Question E was only applied to task 2 results because its aim is to check the effect of enzyme types.

2 $$\begin{aligned}&\hbox {F}1 \sim \hbox {enzyme}\_\hbox {type} + \hbox {type}\_\hbox {padding} \end{aligned}$$

3 $$\begin{aligned}&\hbox {F1} \sim \hbox {architecture} + \hbox {enzyme}\_\hbox {type} + \hbox {type}\_\hbox {padding}\nonumber \\&\quad + \hbox {type}\_\hbox {padding:architecture} \end{aligned}$$

4 $$\begin{aligned}&\hbox {F}1 \sim \hbox {architecture} + \hbox {enzyme}\_\hbox {type} + \hbox {type}\_\hbox {padding} \nonumber \\&\quad + \hbox {type}\_\hbox {padding:enzyme}\_\hbox {type} \end{aligned}$$

**Table 3:** The explanatory model for each question is specified by the column Equation. The Architecture column and the Padding type column show the architectures and padding types included in each comparison, respectively. All the enzyme types are included for each question. Reference categories are indicated in bold.

Questions on the effect of padding addressed through the explanatory models.				
	Question	Equation	Architecture	Padding type
A	Does padding position affect performance?	Eq. 2	**only_denses**	**post-**, pre-, mid-, ext-, strf-, rnd-, zoom-, aug-
B	What is the effect of switching between dense paddings?	Eq. 2	**stack_conv**	**post-**, pre-, mid-, ext-
C	What is the effect of changing from the standard dense padding to sparse padding?	Eq. 2	**stack_conv**	**post-**, strf-, rnd-, zoom-
D	Is an ensemble of paddings beneficial?	Eq. 3	**stack_conv** 1_conv only_denses	**aug-**, post-, strf-
E	Does the effect of padding type on performance depends on the enzyme type?	Eq. 4	**stack_conv** 1_conv only_denses	**post-**, strf-

Linear models were built in the R statistical programming language^49^. *P* values were adjusted for multiple testing by the False Discovery Rate (FDR) by Benjamini-Hochberg^50^.

## Supplementary information

Supplementary Information.

## Data Availability

The UniprotKB/Swiss-Prot database (version 2019_05) protein entries analysed during the current study can be accessed and downloaded through the following link: http://ftp.ebi.ac.uk/pub/databases/uniprot/previous_releases/release-2019_05/knowledgebase/uniprot_sprot-only2019_05.tar.gz . Since this data needs further filtering to get only taxonomy Archaea, we have also uploaded data analysed in this article to the following repository: 10.6084/m9.figshare.11985750. The code is publicly available at https://github.com/b2slab/padding_benchmark.
