# Supplementary material for: Effect of sequence padding on the performance of deep learning models in archaeal protein functional prediction
Source: Sci Rep. 2020 Sep 3;10:14634. doi: 10.1038/s41598-020-71450-8 (PMC7471694; doi:10.1038/s41598-020-71450-8)
Supplement: Supplementary file 1 — Supplementary Information. [file 41598_2020_71450_MOESM1_ESM.pdf]

Supporting Information

Effect of Sequence Padding on the Performance of Deep Learning Models in Archaeal Protein Functional Prediction

Angela Lopez-del Rio[*,1,2], Maria Martin[3], Alexandre Perera-Lluna[1,2], and Rabie Saidi[3]

[1]B2SLab, Departament d'Enginyeria de Sistemes, Automàtica i Informàtica Industrial, Universitat Politècnica de Catalunya, 08028 Barcelona, Spain.
[2]Department of Biomedical Engineering, Institut de Recerca Pediàtrica Hospital Sant Joan de Dèu, Esplugues de Llobregat, 08950, Spain.
[3]European Molecular Biology Laboratory, European Bioinformatics Institute (EMBL-EBI), Hinxton, CB10 1SD,UK.

E-mail: angela.lopez.del.rio@upc.edu

1 Results

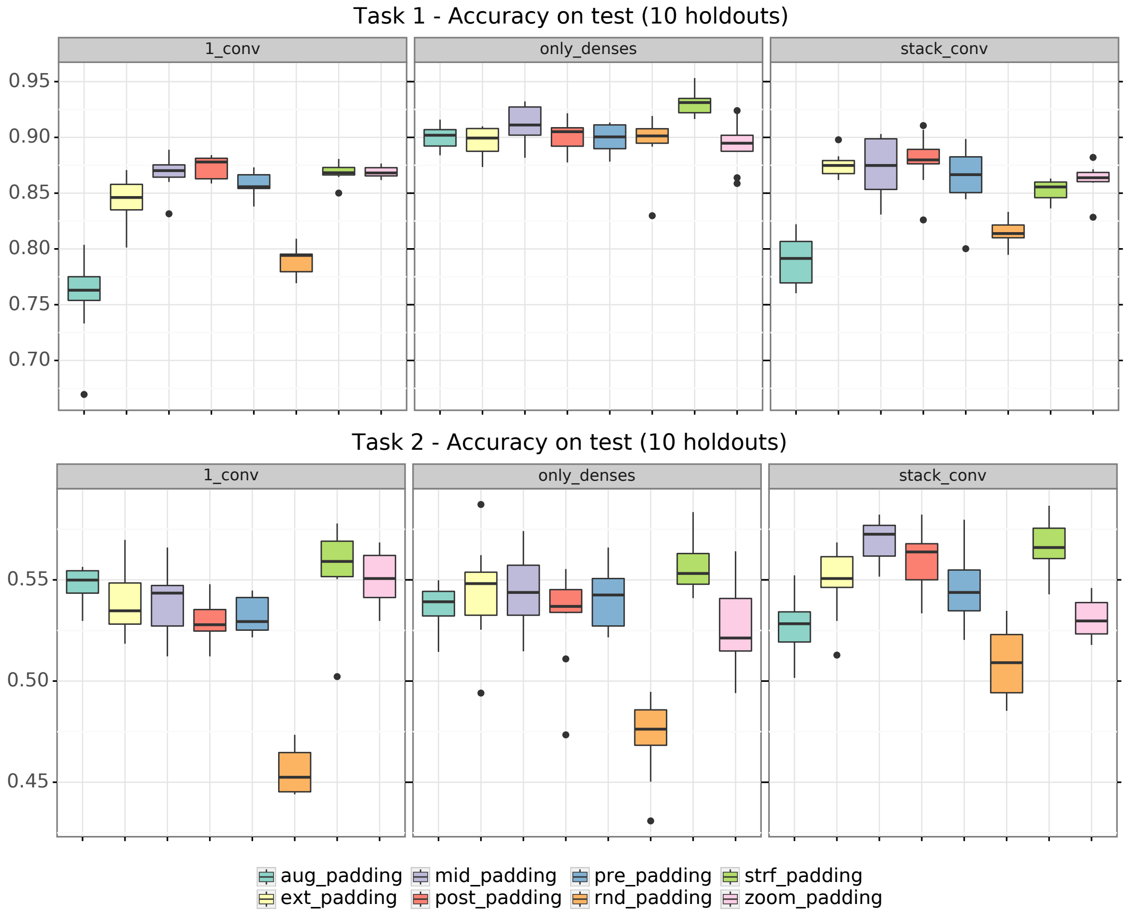

Figure S1: Accuracy on test distributions for each type of padding in each tested architecture

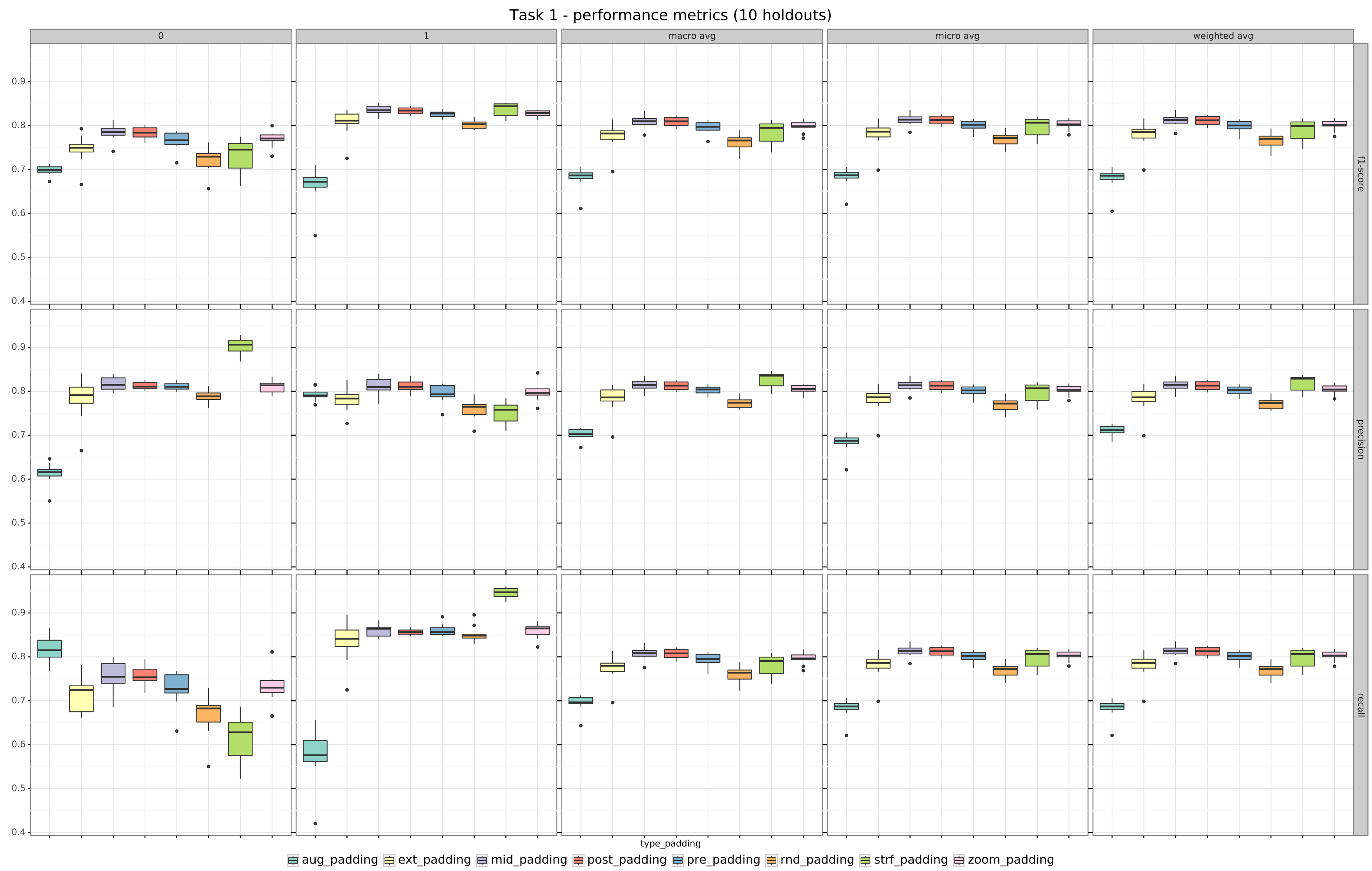

Figure S2: Task 1 F1-score measurements detailed for each label (0: non-enzyme, 1: enzyme) **only_denses** architecture.

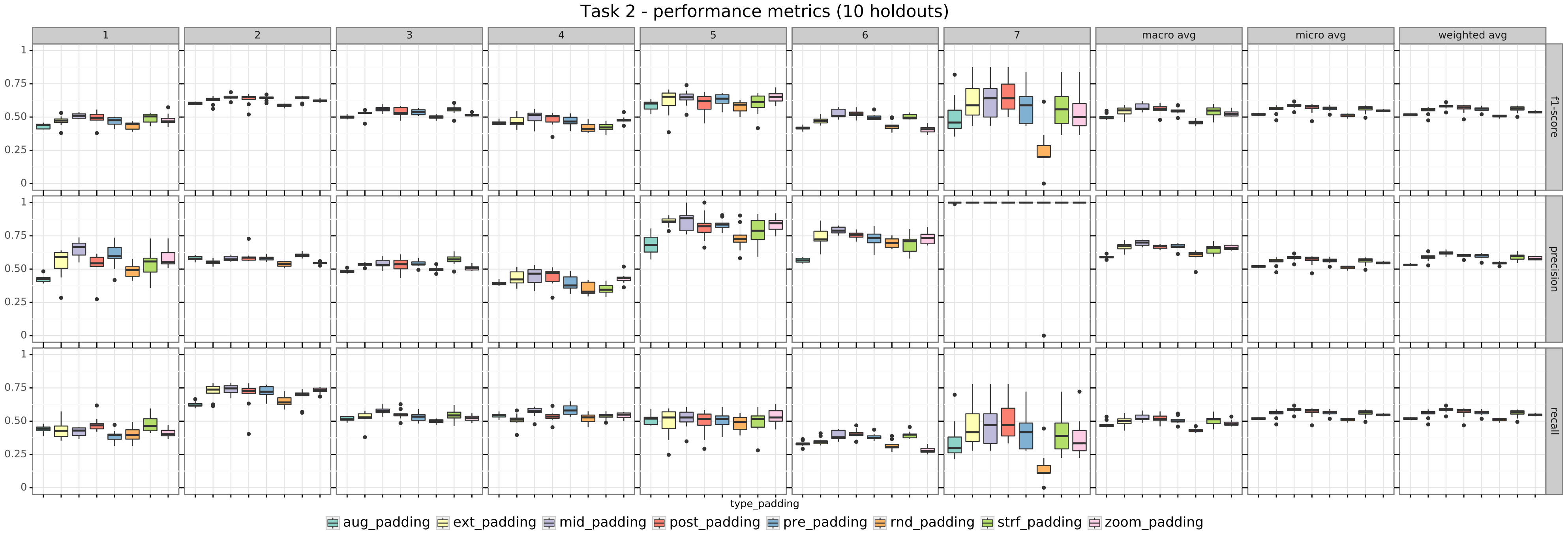

Figure S3: Task 2 F1-score measurements detailed for each label (1-7: enzyme classes) in **only_denses** architecture.

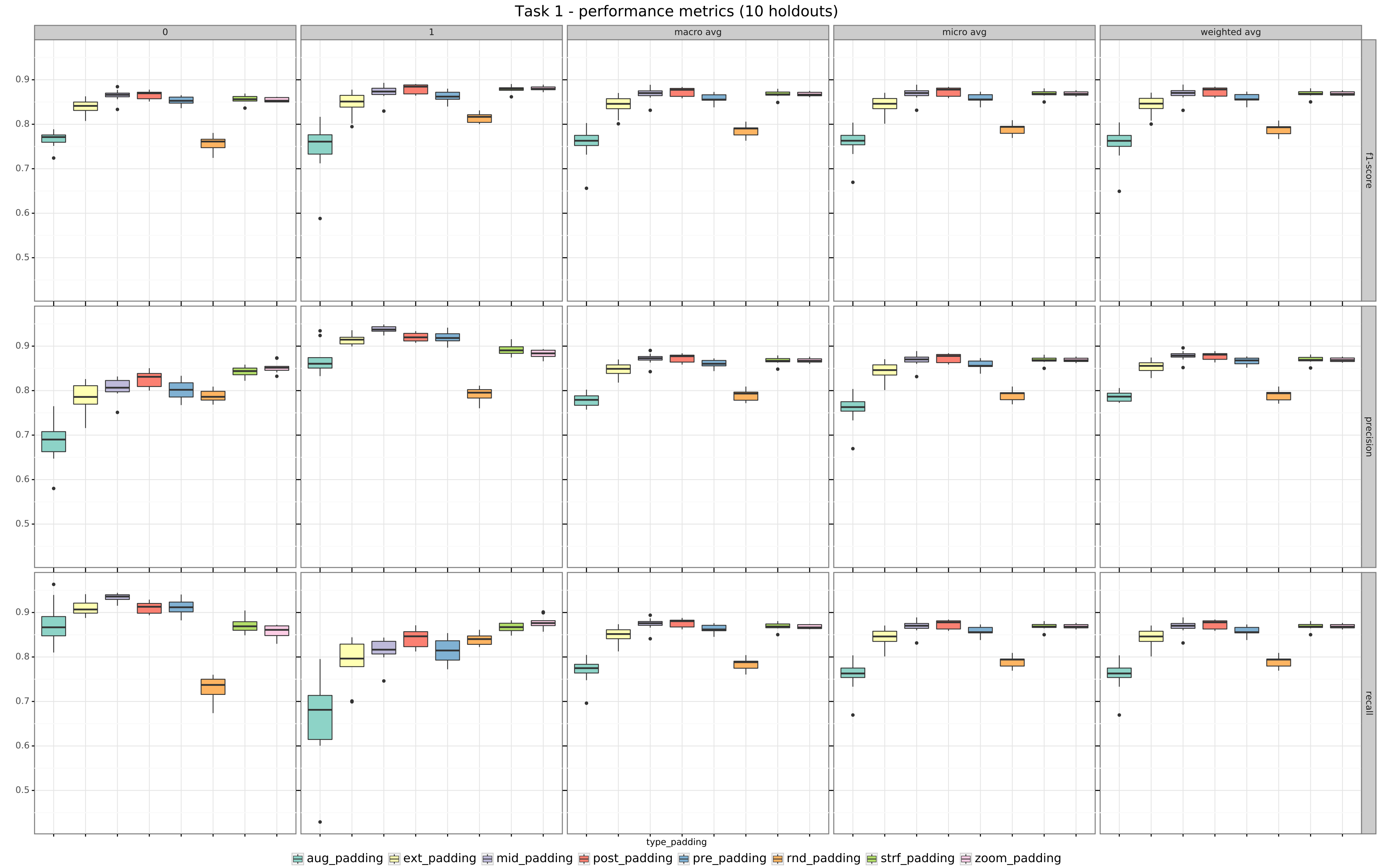

Figure S4: Task 1 F1-score measurements detailed for each label (0: non-enzyme, 1: enzyme) in **1_conv** architecture.

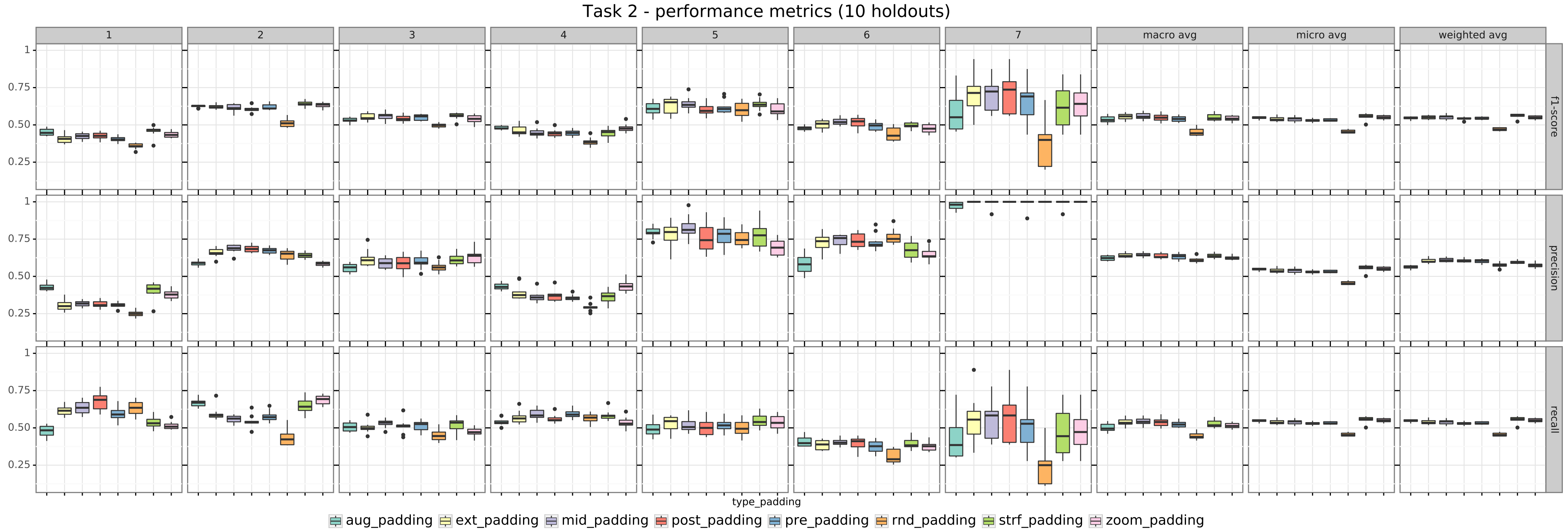

Figure S5: Task 2 F1-score measurements detailed for each label (1-7: enzyme classes) in **1_conv** architecture.

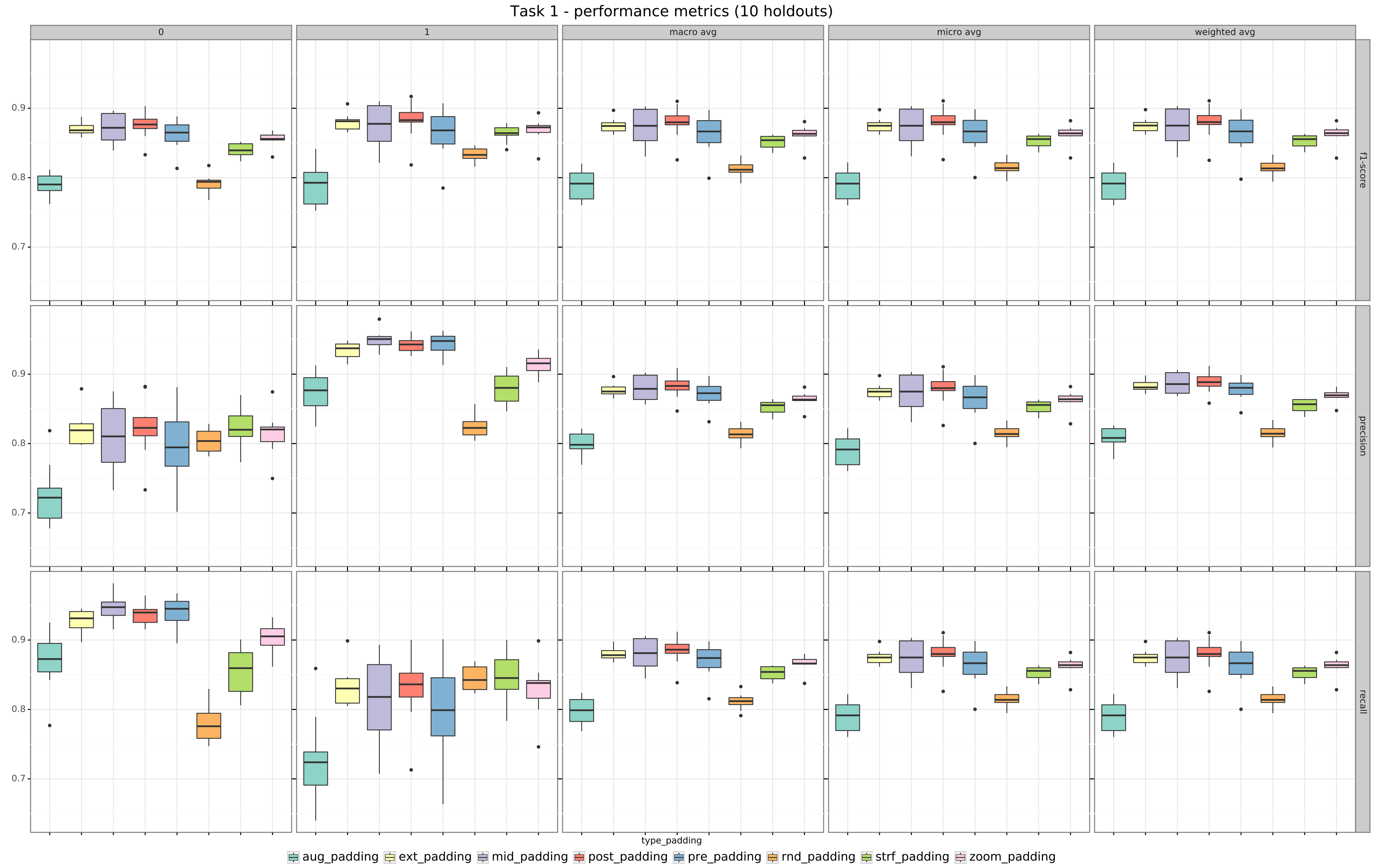

Figure S6: Task 1 F1-score measurements detailed for each label (0: non-enzyme, 1: enzyme) in **stack_conv** architecture.

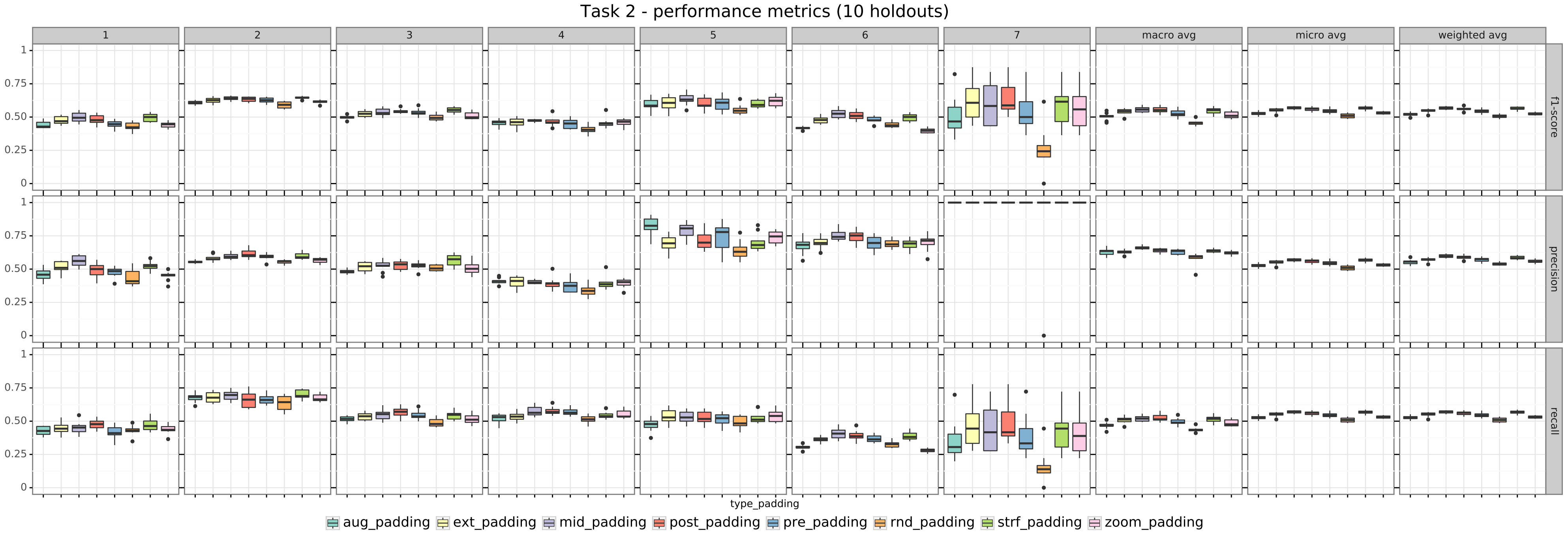

Figure S7: Task 2 F1-score measurements detailed for each label (1-7: enzyme classes) in **stack_conv** architecture.

All linear models tables have been created with stargazer v.5.2.2 [1].

Table S1: Full additive linear models on F1-score. The reference levels were omitted.

	Task 1	Task 2
architecture1_conv	−0.063*** (−0.069, −0.057)	0.021*** (0.011, 0.030)
architecturestack_conv	−0.052*** (−0.058, −0.046)	0.010** (0.0004, 0.020)
enz_type1	0.012*** (0.008, 0.017)	
enz_type2		0.167*** (0.152, 0.182)
enz_type3		0.097*** (0.082, 0.111)
enz_type4		0.008 (−0.007, 0.023)
enz_type5		0.167*** (0.152, 0.182)
enz_type6		0.044*** (0.029, 0.059)
enz_type7		0.100*** (0.085, 0.115)
type_paddingaug_padding	−0.069*** (−0.078, −0.059)	−0.014* (−0.029, 0.002)
type_paddingext_padding	−0.013*** (−0.023, −0.003)	0.008 (−0.008, 0.024)
type_paddingmid_padding	0.0001 (−0.010, 0.010)	0.015* (−0.001, 0.031)
type_paddingpre_padding	−0.011** (−0.020, −0.001)	−0.001 (−0.017, 0.015)
type_paddingrnd_padding	−0.053*** (−0.062, −0.043)	−0.085*** (−0.101, −0.069)
type_paddingstrf_padding	−0.001 (−0.011, 0.009)	0.011 (−0.005, 0.027)
type_paddingzoom_padding	−0.010* (−0.019, −0.00001)	−0.033*** (−0.049, −0.017)
Intercept	0.916*** (0.908, 0.924)	0.444*** (0.428, 0.460)
Observations	480	1,680
R^2	0.666	0.427
Adjusted R^2	0.659	0.422
Residual Std. Error	0.027 (df = 469)	0.083 (df = 1664)
F Statistic	93.390*** (df = 10; 469)	82.834*** (df = 15; 1664)
Note:		*p<0.1; **p<0.05; ***p<0.01

Table S2: Linear model on F1-score to analyze if padding position affects performance. The reference levels were omitted.

	Task 1	Task 2
enz_type1	0.006 (−0.005, 0.016)	
enz_type2		0.157*** (0.130, 0.184)
enz_type3		0.060*** (0.033, 0.087)
enz_type4		−0.013 (−0.040, 0.014)
enz_type5		0.136*** (0.109, 0.163)
enz_type6		0.024* (−0.003, 0.051)
enz_type7		0.125*** (0.098, 0.152)
type_paddingext_padding	−0.004 (−0.018, 0.010)	−0.014 (−0.034, 0.007)
type_paddingmid_padding	−0.006 (−0.020, 0.009)	0.004 (−0.017, 0.024)
type_paddingpre_padding	−0.017** (−0.031, −0.002)	−0.027** (−0.047, −0.006)
Intercept	0.876*** (0.865, 0.888)	0.484*** (0.461, 0.507)
Observations	80	280
R^2	0.083	0.538
Adjusted R^2	0.034	0.523
Residual Std. Error	0.023 (df = 75)	0.062 (df = 270)
F Statistic	1.697 (df = 4; 75)	34.933*** (df = 9; 270)
Note:		*p<0.1; **p<0.05; ***p<0.01

Table S3: Linear model on F1-score to analyze what is the effect of switching between dense paddings. The reference levels were omitted.

	Task 1	Task 2
enz_type1	0.006 (−0.005, 0.016)	
enz_type2		0.157*** (0.130, 0.184)
enz_type3		0.060*** (0.033, 0.087)
enz_type4		−0.013 (−0.040, 0.014)
enz_type5		0.136*** (0.109, 0.163)
enz_type6		0.024* (−0.003, 0.051)
enz_type7		0.125*** (0.098, 0.152)
type_paddingext_padding	−0.004 (−0.018, 0.010)	−0.014 (−0.034, 0.007)
type_paddingmid_padding	−0.006 (−0.020, 0.009)	0.004 (−0.017, 0.024)
type_paddingpre_padding	−0.017** (−0.031, −0.002)	−0.027** (−0.047, −0.006)
Intercept	0.876*** (0.865, 0.888)	0.484*** (0.461, 0.507)
Observations	80	280
R^2	0.083	0.538
Adjusted R^2	0.034	0.523
Residual Std. Error	0.023 (df = 75)	0.062 (df = 270)
F Statistic	1.697 (df = 4; 75)	34.933*** (df = 9; 270)
Note:		*p<0.1; **p<0.05; ***p<0.01

Table S4: Linear model on F1-score to analyze what is the effect of changing from the standard dense padding to sparse paddings. The reference levels were omitted.

	Task 1	Task 2
enz_type1	0.022*** (0.014, 0.029)	
enz_type2		0.158*** (0.125, 0.192)
enz_type3		0.064*** (0.030, 0.097)
enz_type4		−0.015 (−0.048, 0.018)
enz_type5		0.132*** (0.099, 0.165)
enz_type6		−0.001 (−0.034, 0.032)
enz_type7		0.050*** (0.016, 0.083)
type_paddingrnd_padding	−0.067*** (−0.078, −0.056)	−0.098*** (−0.123, −0.073)
type_paddingstrf_padding	−0.028*** (−0.039, −0.017)	−0.007 (−0.032, 0.018)
type_paddingzoom_padding	−0.017*** (−0.028, −0.006)	−0.038*** (−0.063, −0.013)
Intercept	0.868*** (0.860, 0.877)	0.498*** (0.470, 0.526)
Observations	80	280
R^2	0.720	0.497
Adjusted R^2	0.705	0.481
Residual Std. Error	0.017 (df = 75)	0.076 (df = 270)
F Statistic	48.257*** (df = 4; 75)	29.692*** (df = 9; 270)
Note:		*p<0.1; **p<0.05; ***p<0.01

Table S5: Linear model on F1-score to analyze if an ensemble of paddings is beneficial. The reference levels were omitted.

	Task 1	Task 2
architecture1_conv	−0.034*** (−0.048, −0.020)	0.032** (0.007, 0.057)
enz_type2		0.166*** (0.144, 0.188)
enz_type3		0.093*** (0.071, 0.115)
enz_type4		0.003 (−0.019, 0.025)
enz_type5		0.154*** (0.132, 0.176)
enz_type6		0.039*** (0.016, 0.061)
enz_type7		0.124*** (0.102, 0.146)
architectureonly_denses	0.110*** (0.096, 0.123)	0.027** (0.002, 0.052)
enz_type1	0.009*** (0.003, 0.015)	
type_paddingpost_padding	0.089*** (0.076, 0.103)	0.050*** (0.025, 0.075)
type_paddingstrf_padding	0.061*** (0.048, 0.075)	0.043*** (0.018, 0.068)
architecture1_conv:type_paddingpost_padding	0.028*** (0.008, 0.047)	−0.036** (−0.071, −0.0004)
architectureonly_denses:type_paddingpost_padding	−0.089*** (−0.108, −0.070)	−0.072*** (−0.108, −0.037)
architecture1_conv:type_paddingstrf_padding	0.050*** (0.031, 0.069)	−0.029 (−0.064, 0.007)
architectureonly_denses:type_paddingstrf_padding	−0.031*** (−0.050, −0.012)	−0.026 (−0.061, 0.010)
Intercept	0.786*** (0.775, 0.796)	0.421*** (0.399, 0.444)
Observations	180	630
R^2	0.859	0.444
Adjusted R^2	0.851	0.431
Residual Std. Error	0.022 (df = 170)	0.076 (df = 615)
F Statistic	114.720*** (df = 9; 170)	35.043*** (df = 14; 615)
Note:		*p<0.1; **p<0.05; ***p<0.01

Table S6: Linear model on F1-score to analyze how might enzyme type affect differently the performances of some padding types. The reference levels were omitted.

	Task 1	Task 2
architecture1_conv	0.005 (−0.003, 0.013)	−0.0002 (−0.019, 0.019)
enz_type2		0.162*** (0.120, 0.204)
enz_type3		0.098*** (0.056, 0.140)
enz_type4		−0.003 (−0.044, 0.039)
enz_type5		0.141*** (0.100, 0.183)
enz_type6		0.075*** (0.033, 0.116)
enz_type7		0.140*** (0.099, 0.182)
architectureonly_denses	0.050*** (0.042, 0.058)	−0.022** (−0.041, −0.002)
enz_type1	0.009* (−0.001, 0.018)	
type_paddingstrf_padding	−0.006 (−0.015, 0.003)	0.030 (−0.012, 0.072)
enz_type1:type_paddingstrf_padding	0.010 (−0.004, 0.023)	
enz_type2:type_paddingstrf_padding		−0.006 (−0.065, 0.053)
enz_type3:type_paddingstrf_padding		−0.009 (−0.068, 0.050)
enz_type4:type_paddingstrf_padding		−0.036 (−0.095, 0.023)
enz_type5:type_paddingstrf_padding		0.001 (−0.058, 0.059)
enz_type6:type_paddingstrf_padding		−0.047 (−0.106, 0.011)
enz_type7:type_paddingstrf_padding		−0.034 (−0.092, 0.025)
Intercept	0.861*** (0.853, 0.869)	0.457*** (0.426, 0.489)
Observations	120	420
R^2	0.629	0.405
Adjusted R^2	0.613	0.383
Residual Std. Error	0.019 (df = 114)	0.082 (df = 404)
F Statistic	38.674*** (df = 5; 114)	18.319*** (df = 15; 404)
Note:		*p<0.1; **p<0.05; ***p<0.01

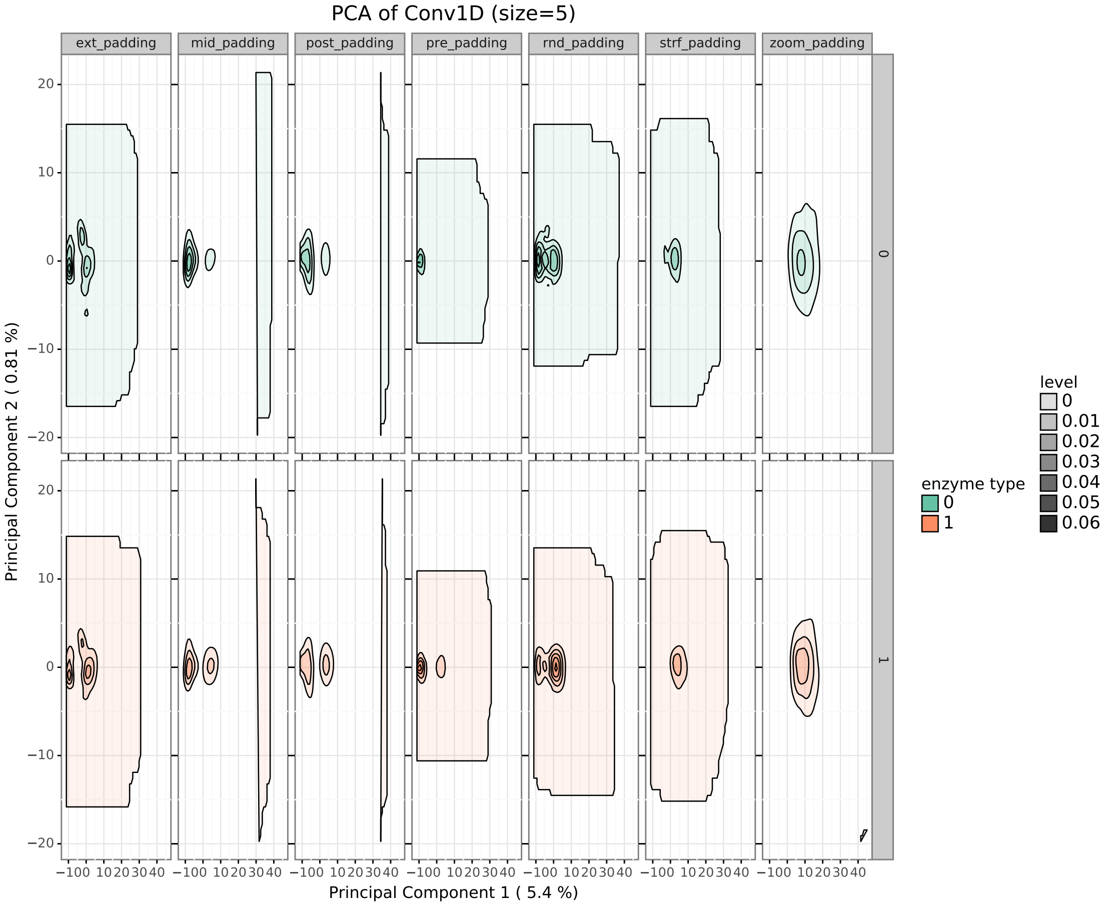

Figure S8: Density representation of PC1 vs PC2 of the activations of the 1D Convolutional layer of1conv model for each type of padding in task 1.

In Figure S8, activations seem to be more spread along the PC space than in Figure 3, the analogous representation for task 2. This happens for all types of padding except for zoom-, whose distribution is very similar to that of zoom-padding for task 2.

In order to quantify the differences observed in the first principal component of the activations of the convolutional filters for task 2 models, we built the explanatory model explained by Equation 1. Results can be seen in Table S7. All terms are statistically significant, which means there are differences for PC1 according to enzyme type and padding type. Terms for enzyme types 2, 3 and 4 are negative while they are positive for 1, 5, 6 and 7.

$$\text{PC1} \sim \text{enzyme\_type} + \text{type\_padding} \tag{1}$$

Table S7: Linear model on PC1 of the activations. The reference levels were omitted.

paddingext_padding	−0.007 (−0.271, 0.258)
paddingmid_padding	−0.524*** (−0.789, −0.259)
paddingpre_padding	−1.181*** (−1.445, −0.916)
paddingrnd_padding	6.355*** (6.091, 6.620)
paddingstrf_padding	7.893*** (7.628, 8.158)
paddingzoom_padding	6.473*** (6.209, 6.738)
enzyme_type2	−2.425*** (−2.690, −2.161)
enzyme_type3	−1.305*** (−1.570, −1.041)
enzyme_type4	−1.835*** (−2.100, −1.570)
enzyme_type5	0.960*** (0.695, 1.224)
enzyme_type6	3.128*** (2.864, 3.393)
enzyme_type7	1.582*** (1.318, 1.847)
Intercept	−2.731*** (−2.986, −2.476)
Observations	62,720
R^2	0.172
Adjusted R^2	0.172
Residual Std. Error	9.041 (df = 62707)
F Statistic	1,085.138*** (df = 12; 62707)
Note:	*p<0.1; **p<0.05; ***p<0.01

2 Data distribution

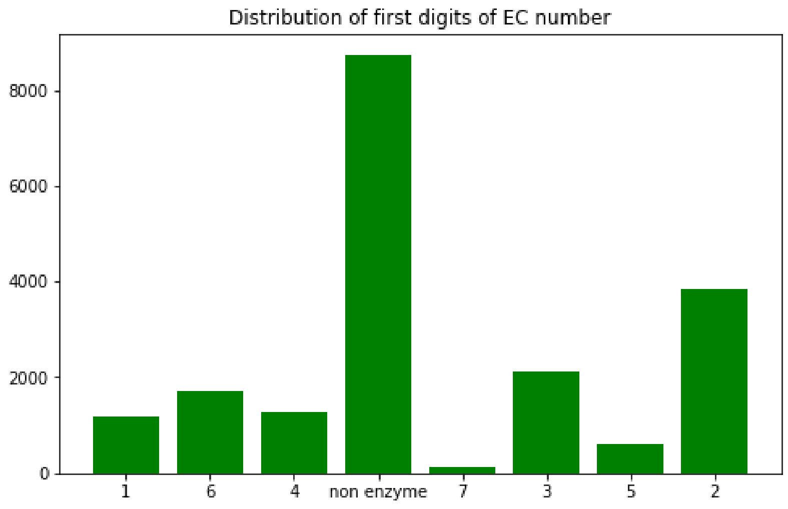

Figure S9: Distribution of enzyme types in the Archaea UniprotKB proteins.

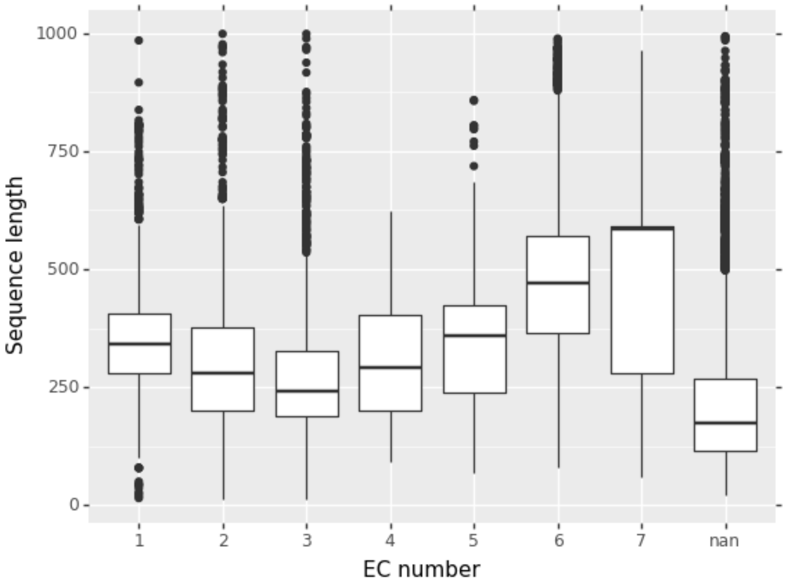

Figure S10: Sequence length stratified by enzyme type (according to the first digit of the EC number). 'nan' refers to proteins without EC number (non-enzymes). p-value = 0.0 for Kruskal-Wallis H-test for independent samples, so sequence length differences between different enzyme types are significant.

3 Models architecture

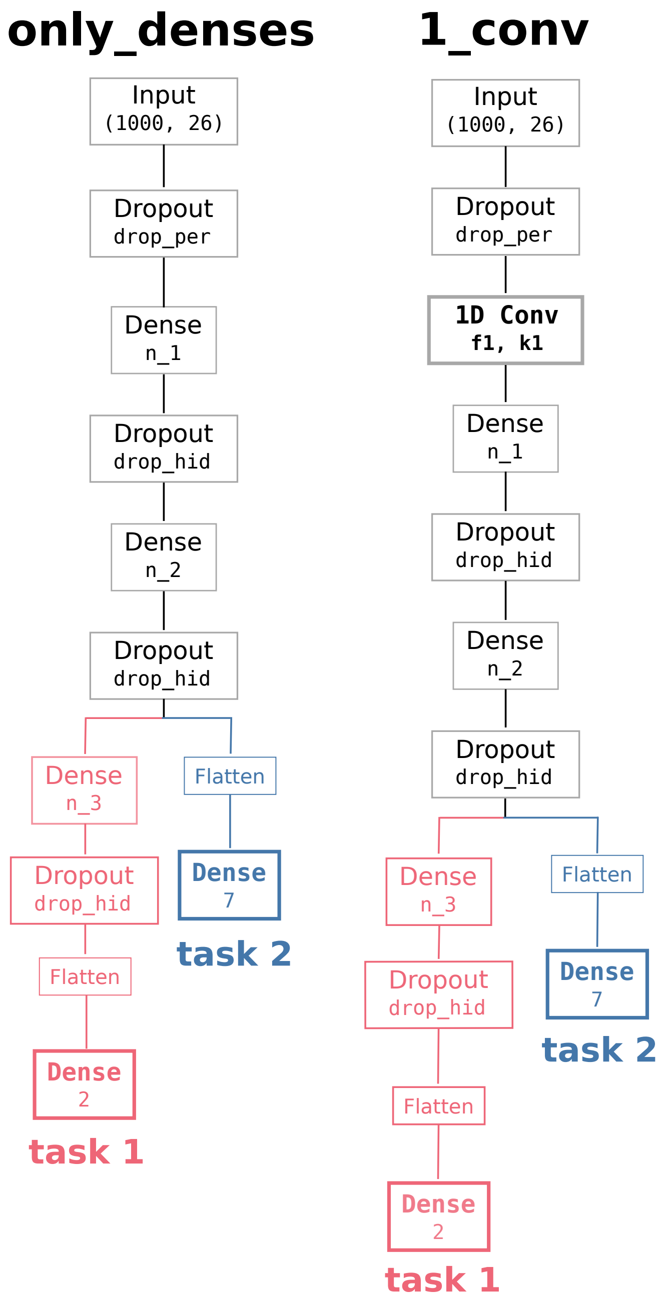

Figure S11: Schematic representation of two of the tested architectures: **only_denses** and **1_conv**. Each one of the architectures comprises two different models: task 1 and task 2.

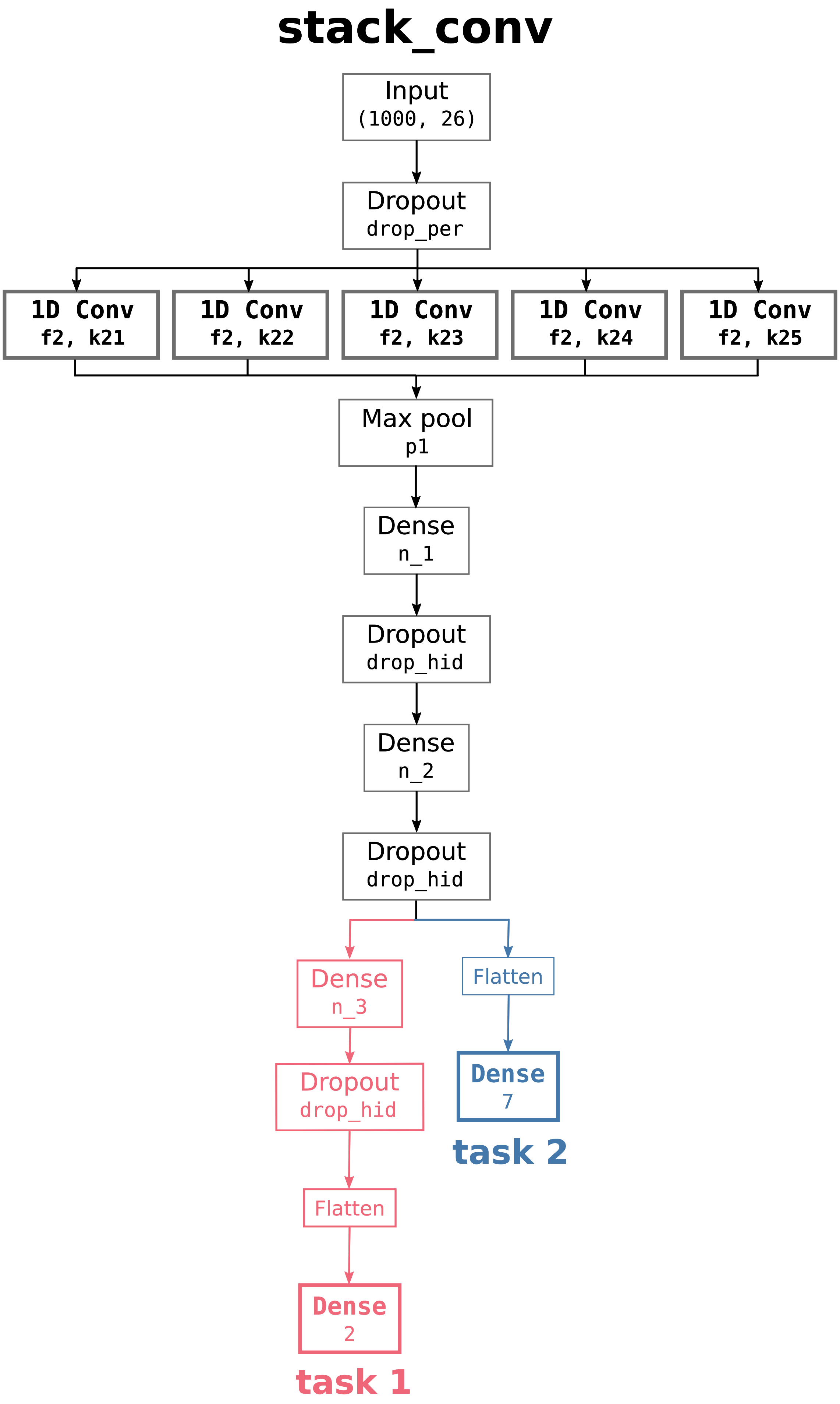

Figure S12: Schematic representation of **stack_conv** architecture. Each one of the architectures comprises two different models: task 1 and task 2.

The selected parameters were drop_per=0.2, drop_hid=0.5 for all the models; n_1=314, n_2=77, n_3=8 for task 1 and n_1=313, n_2=76 for task 2; pool_size=10 for stack_conv; f1=64 and k1=5 for 1_conv; f2=10, k21=1, k22=3, k23=5, k24=9, k25=15 for stack_conv. As activation function, we used Rectified Linear Unit (ReLU) for the hidden layers [2], and *softmax* for the output layers.

The set of values for the number of neurons in the feed-forward part of the model, which is common for the four architectures, is based on the number of enzyme classes and subclasses. There are 7 classes of enzymes according to the first digit of the EC number (8 if we take count non-enzymes), 76 subclasses according to the second digit of the EC number (77 with non-enzymes) and 313 categories according to the third digit of the EC number (314 with the non-enzymes). So the values for the feed-forward layers would be 314, 77 and 8, respectively. These values aim to have a biological meaning since deep neural networks are able to extract hierarchical feature representations and enzyme classification has a tree-structured label space.

4 Performance metrics

The description of the metrics used for evaluating and comparing the performance of the different padding types is shown below. Let TP be the number of true positive classified samples, TN the true negatives, FP the false positives and FN the false negatives:

$$Accuracy = \frac{(TP + TN)}{(TP + FP + TN + FN)}$$

.

If $precision = \frac{TP}{TP+FP}$ and $recall = \frac{TP}{TP+FN}$, then F1-score can be described as

$$F_1 = 2 \cdot \frac{precision \cdot recall}{precision + recall}$$

The macro F1-score calculates metrics for each label, and finds their unweighted mean. This does not take label imbalance into account.

The Area Under the Curve (AUC) of the Receiver Operating Characteristic (ROC) curve measures performance in classification problems for different thresholds. The ROC curve is a probability curve obtained by plotting the True Positive Rate (which is the same as the recall) on y-axis against the False Positive Rate (which is $\frac{FP}{TN+FP}$) on the x-axis. The AUC, which is the area under this curve, quantifies how capable the model is of distinguishing both classes.

References

[1] Hlavac M. stargazer: Well-Formatted Regression and Summary Statistics Tables. Bratislava, Slovakia; 2018. R package version 5.2.2. Available from: https://CRAN.R-project.org/package=stargazer.

[2] Lecun Y, Bengio Y, Hinton G. Deep learning. Nature. 2015;521(7553):436–444.
